# Diagnosis of tuberculosis in children: increased need for better methods.

**DOI:** 10.3201/eid0104.950402

**Published:** 1995

**Authors:** E A Khan, J R Starke

**Affiliations:** Baylor Collge of Medicine, Houston, Texas, USA. jstarke@msmailpo2.is5.tch.tmc.edu

## Abstract

In the last decade tuberculosis (TB) has reemerged as a major worldwide public health hazard with increasing incidence among adults and children. Although cases among children represent a small percentage of all TB cases, infected children are a reservoir from which many adult cases will arise. TB diagnosis in children usually follows discovery of a case in an adult, and relies on tuberculin skin testing, chest radiograph, and clinical signs and symptoms. However, clinical symptoms are nonspecific, skin testing and chest radiographs can be difficult to interpret, and routine laboratory tests are not helpful. Although more rapid and sensitive laboratory testing, which takes into account recent advances in molecular biology, immunology, and chromatography, is being developed, the results for children have been disappointing. Better techniques would especially benefit children and infants in whom early diagnosis is imperative for preventing progressive TB.


### Diagnosis of Tuberculosis in Children: Increased Need for Better Methods

**Ejaz A. Khan, M.D., and Jeffrey R. Starke, M.D.**
Baylor College of Medicine, Houston, Texas, USA

In the last decade tuberculosis (TB) has reemerged as a major worldwide public health hazard with increasing incidence among adults and children. Although cases among children represent a small percentage of all TB cases, infected children are a reservoir from which many adult cases will arise. TB diagnosis in children usually follows discovery of a case in an adult, and relies on tuberculin skin testing, chest radiograph, and clinical signs and symptoms. However, clinical symptoms are nonspecific, skin testing and chest radiographs can be difficult to interpret, and routine laboratory tests are not helpful. Although more rapid and sensitive laboratory testing, which takes into account recent advances in molecular biology, immunology, and chromatography, is being developed, the results for children have been disappointing. Better techniques would especially benefit children and infants in whom early diagnosis is imperative for preventing progressive TB.

Despite the availability of effective preventive measures and chemotherapy, the prevalence of tuberculosis (TB) is increasing in the developing world and in much of the industrialized world as well (1-4). According to World Health Organization (WHO) estimates, in 1990 there were 8 million new cases of TB and 3 million deaths due to the disease worldwide; 1.3 million new cases and 450,000 deaths were among children under 15 years of age (5). WHO projects that 90 million new cases and 30 million deaths–including 4.5 million deaths among children–will occur in the 1990s (6,7). In developing countries, the risk for TB infection and disease is relatively uniform in the population; annual rates of infection often exceed 2% (5,6). In industrialized countries, risk is more uneven and depends on the individual's past or present activities and exposure to persons at high risk for the disease (Table 1). From 1987 to 1991, the number of TB cases among children under 5 years of age in the United States increased by 49% from 674 cases to 1006 (8). Although cases among children represent a small percentage of all TB cases, infected children are a reservoir from which many adult cases will arise. The risk for infection by *Mycobacterium tuberculosis* among children depends primarily on the level of risk of developing infectious TB for the adults in their immediate environment, especially their household. Because most current diagnostic tests for TB infection and disease have low specificity and therefore low positive predictive values, epidemiologic investigation continues to be important in establishing the diagnosis of TB in children. In industrialized countries, clinicians and public health professionals in TB services must always ask: Has the child been exposed to an adult with infectious pulmonary TB?

Address for correspondence: Jeffrey R. Starke, Texas Children's Hospital, MC 3-2371, 1102 Bates Street, Houston, TX 77030, USA; fax: 713-770-4347; e-mail jstarke@msmailpo2.is5.tch.tmc.edu.

### Natural History of TB in Children

The natural history of TB in children follows a continuum; however, it is useful to consider three basic stages: exposure, infection, and disease (1). Exposure implies that the child has had recent and substantial contact with an adult or adolescent who has suspected or confirmed contagious pulmonary TB (a source case). Exposed children are usually identified during followup investigations for persons with suspected pulmonary TB by public health workers (9); the child's tuberculin skin test (TST) is nonreactive, the results of the chest radiograph are normal, and the child is free of physical signs or symptoms of TB. Some exposed children are infected with *M. tuberculosis*. The clinician cannot know immediately which exposed children are infected because the development of delayed-type hypersensitivity to tuberculin may take up to 3 months. Unfortunately, in children under 5 years of age, severe TB—especially meningeal and disseminated

Table 1. Persons at high risk for *Mycobacterium tuberculosis* infection in industrialized countries

Persons likely to be exposed to or become infected with *M. tuberculosis*

- Close contacts of a person with infectious tuberculosis (TB)
- Foreign-born persons from high-incidence areas (e.g., Asia, Africa, Latin America)
- The elderly
- Residents of long-term care facilities (e.g., correctional facilities and nursing homes)
- Persons who inject drugs
- Other groups identified locally as having increased prevalence of TB (e.g., migrant farm workers or homeless persons)
- Persons who may have occupational exposure to TB

Persons at high risk of developing TB disease once infected

- Persons recently infected with *M. tuberculosis* (within the past 2 years)
- HIV-infected persons
- Persons with immunosuppressing conditions or medication use
- Persons with a history of inadequately treated TB
- Infants disease—can occur in fewer than 3 months, before the TST becomes reactive (10). Young children in the exposure stage should receive chemotherapy, usually isoniazid, until infection can been excluded.

TB infection is first signaled by a reactive Mantoux TST. In this stage, there are no signs or symptoms, and the results of the chest radiograph are either normal or show only fibrotic lesions or calcifications in the lung parenchyma or regional lymph nodes. In developing countries, TB infection is rarely discovered and almost never treated. In most industrialized countries, children with a positive TST receive isoniazid for 6 to 12 months.

TB disease occurs when signs and symptoms or radiographic manifestations caused by *M. tuberculosis* appear. Radiographic abnormalities and clinical manifestations in infected children probably are influenced by the host inflammatory reaction more than by the number of organisms. Studies show that in 40% to 50% of infants with untreated TB infection disease develops within 1 to 2 years (11). The risk decreases to 15% among older children. In 25% to 35% of children TB is extrapulmonary and more difficult to confirm bacteriologically.

In adults, the distinction between TB infection and disease is usually clear because most disease is caused by reactivation of dormant organisms years after infection. Disease in adults is usually accompanied by symptoms, and patients frequently are infectious. In children, who most often have primary disease, the interval between infection and disease can be several months to several years, and radiographic abnormalities often are not accompanied by symptoms; moreover, these children are rarely infectious. The major reason for separating infection from disease in children is that the perception affects the approach to treatment: infection is generally treated with a single anti-TB drug, whereas active disease is treated with two or more drugs. The rationale for the difference in treatment is that the likelihood of emergence of resistance to a drug increases as the bacillary population increases (3). This distinction is somewhat artificial in children since infection and primary disease are parts of a continuum. Because anti-TB medications are well tolerated by children and are relatively inexpensive in industrialized countries, the usual paradigm of infection and disease encourages overtreatment rather than undertreatment. Asymptomatic lymphadenopathy and mild lung parenchymal changes are labeled and treated as disease.

When evaluating new diagnostic tests the basic differences between the pathophysiology of TB in adults and children should be considered. Among children with recent TB infection, active multiplication of mycobacteria occurs with or without the presence of radiographic abnormalities or clinical symptoms. For example, gastric aspirate cultures yield *M. tuberculosis* from a small proportion of recently infected children with normal chest radiographs. One can anticipate that most diagnostic tests designed to detect *M. tuberculosis* in adults with TB disease will be positive in some proportion of children who have what is usually called TB infection. It will take careful consideration and investigation to determine if and how the results of these new tests should influence the definitions and treatment of TB infection and disease in children.

### Established Diagnostic Methods

### Tuberculin Skin Test (TST)

The Mantoux TST, which uses five tuberculin units of purified protein derivative, is the standard method for detecting infection by *M. tuberculosis*. The reaction is measured as millimeters of induration after 48 to 72 hours. Since TST is the only way to determine asymptomatic infection by *M. tuberculosis*, the false-negative rate cannot be calculated. A negative TST does not rule out TB disease in a child. Approximately 10% of otherwise normal children with culture-proven TB do not react to tuberculin initially (12,13). Most of these children have reactive skin tests during treatment, which suggests that TB disease contributed to the immunosuppression. In most cases, the anergy occurs to all antigens, but in some cases, reactions to tuberculin are negative but reactions to other antigens remain positive (13). The rate of false-negative TST is higher in those who are tested soon after becoming infected with severe TB; in children with debilitating or immunosuppressive illnesses, malnutrition, or viral and certain bacterial infections; in infants; and if poor technique is used (1,14). The rate of false-negative TST in children with TB who are infected with human immunodeficiency virus (HIV) is unknown, but it is certainly higher than 10%.

False-positive reactions to TST are often attributed to asymptomatic infection by environmental nontuberculous mycobacteria (NTM). Vaccination with *M. bovis* can cause transient reactivity to a subsequent TST, but the association is weaker than commonly recognized. Most–80% to 90% in several studies–children who received BCG as infants have a nonreactive TST at 5 years of age (15-17). Among older children or adolescents who receive BCG, most develop a reactive skin test initially; however, by 10 to 15 years postvaccination, 80% to 90% have lost tuberculin reactivity (18,19). Skin test reactivity can be boosted, probably by antigenic stimulation, by serial testing in many children and adults who received BCG (20). Various factors determine the TST reaction size after receipt of BCG (1). Many recipients of BCG have a reactive TST because they are infected with *M. tuberculosis* and are at risk for disease, especially if they have had recent contact with an infectious TB patient (18). In general, TST reaction should be interpreted in the same manner for persons who have received BCG (20) and for unvaccinated persons.

The relatively low sensitivity and specificity of TST make the test very useful for persons at high risk for TB infection or disease but undesirable for use in persons at low risk (21,22). The predictive values of TST can be improved by varying the size of induration considered positive according to epidemiologic risk factors for infection (Table 2). However, most of even 15-mm reactions in children at low risk are false-positive results, and testing of persons at low risk should be discouraged. Although the scheme in Table 2 is scientifically and mathematically valid, it assumes that the clinician and family are willing and able to develop an accurate history for TB risk factors for children and adults in their environments.

### Clinical Signs and Symptoms

Two scenarios lead the clinician to suspect that a child has TB disease. The first occurs when TB is considered during the differential diagnosis for an ill child. This is a common scenario in the developing world but is less common in developed countries. Infants are more likely to be symptomatic than older children with pulmonary TB. The most common symptoms are cough, fever, wheezing, and failure to gain weight (13). Clinical

Table 2. Cut-off size of reactive area for a positive Mantoux tuberculin reaction

| ≥5 mm | ≥10 mm | ≥ 15 mm |
|---|---|---|
| Persons who had contact with infectious persons | Foreign-born persons from high-prevalence countries | No risk factors |
| Persons with an abnormal chest radiograph | Residents of prisons, nursing homes, institutions | |
| HIV-infected and other immunosuppressed persons | Persons who inject drugs | |
| | Persons with other medical risk factors | |
| | Health-care workers | |
| | Locally identified populations at high risk | |
| | Children in contact with adults at high risk | |
| | Infants | |

signs are surprisingly meager, but rales and wheezes over the affected lung field are most common. Signs and symptoms of extrapulmonary TB are referable to the involved organ. The sensitivity and specificity of signs and symptoms are extremely low and can lead to both overdiagnosis and underdiagnosis when radiographs and other tests are not available.

The second scenario occurs when evaluating a child who has had significant contact with an adult with suspected or confirmed TB. Usually the TST is applied first and is reactive. A subsequent chest radiograph or physical examination leads to discovery of early disease. The child is usually relatively asymptomatic. In the United States, about 50% of childhood TB cases are discovered in this manner (13).

### Radiologic Studies

Evidence of pulmonary TB in chest radiographs varies (23,24), but usually radiographs show enlargement of hilar, mediastinal, or subcarinal lymph nodes and lung parenchymal changes (Figure 1). Most of the radiographic abnormalities are caused by a combination of lung disease and the mechanical changes induced by partial or complete airway obstruction resulting from enlarging intrathoracic nodes. The most common findings are segmental hyperinflation then atelectasis, alveolar consolidation, interstitial densities, pleural effusion, and, rarely, a focal mass. Cavitation is rare in young children but is more common in adolescents, who may develop reactivation disease similar to that seen in adults.

The development of radiographic techniques, such as computed tomography (CT) scanning, illustrates some of the issues that arise when newer and more sensitive diagnostic tests become available (24). A CT scan may show enlarged or prominent mediastinal or hilar lymph nodes in some children with recent TB infection and a normal chest radiograph (25). In the absence of a CT scan, the child's disease stage would be called TB infection, and single drug therapy would be used. Many studies, involving thousands of children have shown this treatment to be successful. However, when the CT scan shows mild adenopathy, the clinician may consider this finding indicative of TB disease and treat with several drugs, although this probably is not necessary in the absence of drug resistance. These findings reinforce the idea that pediatric TB is a continuum, and the distinction between infectionand disease is somewhat artificial. There is no current role for the CT scan in the evaluation of the asymptomatic TB-infected child with a normal chest radiograph. This scan can be helpful in selected cases to demonstrate endobronchial disease, pericardial invasion, early cavitation, and bronchiectasis resulting from pulmonary TB when the chest radiograph is abnormal but the pathologic process is not clear.

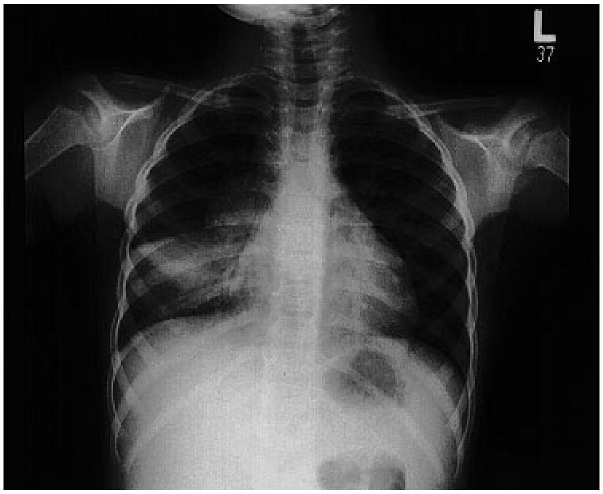

Figure 1. Chest radiograph of a girl with pulmonary tuberculosis. Note the significant hilar adenopathy in association with atelectasis, the so-called collapse-consolidation lesion.

### Mycobacterial Detection and Isolation

Despite recent advances, early mycobacteriologic diagnosis of TB still relies primarily on examination of acid-fast-stained smears from clinical specimens. It is the easiest, least expensive, and most rapid procedure for obtaining preliminary information. However, children under 12 years of age with pulmonary TB rarely produce sputum and are usually unable to expectorate voluntarily. When sputum samples cannot be obtained, gastric aspirate samples are used for detection and isolation of *M. tuberculosis*. Even though an acid-fast bacilli (AFB) stain of sputum is positive in up to 75% of adults with pulmonary TB, fewer than 20% of children with TB have a positive AFB smear of sputum or gastric aspirate (26,27). The newer fluorochrome stains, such as auramine and rhodamine, are superior to classic carbolfuchsin stains (28). The rates of positive AFB stain from body fluids and tissues in children with extrapulmonary TB also are low, and false-positive results caused by NTM disease are common, especially in cervical lymph nodes.

For most children with pulmonary TB, culture confirmation is not needed. Diagnosis is made on the basis of a positive TST, clinical and radiographic findings suggestive of TB, and history of contact with an adult source case. The drug-susceptibility test results from the source case isolate can be used to design the optimal treatment for the child. However, cultures should be obtained from the child if the source patient is unknown or has a drug-resistant organism and if the child is immunocompromised or has extrapulmonary TB.

The best specimen for culture from children with suspected pulmonary TB is the early morning gastric aspirate obtained in the hospital by using a nasogastric tube before the child arises and peristalsis empties the stomach of the respiratory secretions swallowed overnight (29,30). Three consecutive morning gastric aspirates yield *M. tuberculosis* in only 30% to 50% of cases, although the yield from infants is as high as 70% (14). The culture yield from other body fluids or tissues from children with extrapulmonary TB is usually less than 50% (13). Gastric aspiration is inconvenient, expensive, and uncomfortable. The culture yield from random, outpatient gastric aspirates has not been determined recently. Therefore, this procedure cannot be recommended but should be studied.

### Bronchoscopy

The role of bronchoscopy in evaluating children for TB is controversial. The culture yield is lower from bronchoscopy specimens than from properly obtained gastric aspirates (29,31). Most children do not need flexible fiberoptic bronchoscopy, but the procedure may be useful in diagnosing endobronchial TB and excluding other causes of pulmonary abnormality, particularly in immunocompromised children, such as those with HIV infection in whom other opportunistic infections may coexist with or mimic TB. In a recent study of 36 children with pulmonary TB, bronchoscopy showed endobronchial involvement in 42%; most (63%) of these children had no clinical or radiographic evidence of endobronchial TB (31). This technique may be used to determine if a child might benefit from corticosteroid therapy, but guidelines for making this decision have not been established.

### Clinical Scoring System

TB is an enormous problem in developing countries, where about 95% of cases occur (6). Cost, technical difficulties, and lack of resources make TB diagnosis in children very difficult in these countries. Various clinical scoring systems have been proposed on the basis of available information and tests (32,33) (Table 3). Although helpful, many of these systems have low sensitivity and specificity. However, even in industrialized countries, the triad of a positive tuberculin skin test, an abnormal radiograph, and a history of exposure to an adult with TB remains the most effective method for diagnosing TB in children.

### New Diagnostic Techniques

### Polymerase Chain Reaction (PCR)

Diagnostic PCR is a technique of DNA amplification that uses specific DNA sequences as markers for microorganisms (34). In theory, this technique can detect a single organism in a specimen such as sputum, gastric aspirate, pleural fluid, cerebrospinal fluid, or blood. Recent publications show that various PCR techniques, most using the mycobacterial insertion element IS6110 as the DNA marker for *M. tuberculosis*-complex organisms, have a sensitivity and specificity greater than 90% for detecting pulmonary TB in adults (35,36). However, these tests are not performed correctly in all clinical laboratories (36)

Table 3. A set of criteria for the diagnosis of pulmonary tuberculosis (TB) in children when culture is not available

A. Positive acid-fast stain of sputum or gastric aspirate or

B. Two or more of the following:

- History of contact with a tuberculous adult
- Cough lasting longer than 2 weeks
- A reactive tuberculin skin test
  - ≥ 10 mm in children without prior BCG vaccination
  - ≥ 15 mm in children with prior BCG vaccination
- Radiographic findings compatible with TB
- Response to anti-TB therapy (increased body weight by 10% after 2 months, decrease in symptoms)

Source: ref. 33.

and may offer little advantage over high-quality microscopic examination of sputum (34). The cost involved and the need for sophisticated equipment and scrupulous technique to avoid cross-contamination of specimens preclude the use of PCR techniques in many developing countries. PCR may have a special role in the diagnosis of extrapulmonary TB and pulmonary TB in children since sputum smears are usually unrevealing in these cases.

Use of PCR for detecting *M. tuberculosis* in children has not been evaluated extensively. Pierre et al. (37) used an IS6110-based PCR to detect *M. tuberculosis* in gastric aspirate samples from 22 children with pulmonary TB. They found that 15 (25%) of 59 samples were positive; however, testing multiple samples or testing samples at least twice improved the sensitivity. When three samples from the same patient were tested two times each, two or more positive results were obtained from 9 of 15 children with TB, but from 0 of 17 controls. However, 2 of 65 single samples from controls were positive by PCR. Using an IS6110-based PCR assay, Starke et al. (38) tested gastric aspirates from 35 hospitalized children with pulmonary TB and 30 controls to detect *M. tuberculosis*. When compared with the clinical diagnosis, PCR had a sensitivity of 40% and specificity of 80%. Six controls had false-positive PCR results; one had a recent TB infection, two had NTM disease, and three had conditions unrelated to mycobacterial infection. Delacourt et al. (39) studied 199 specimens from 68 children with suspected TB. An IS6110-based PCR identified *M. tuberculosis* in clinical samples from 83% of children with disease compared to the low yield from positive AFB smears (21%) and positive cultures (42%) (39). PCR identified 70% of children with clinical pulmonary TB but no other microbiologic proof of the infection. However, 39% of children with infection but no radiographic or clinical disease also had positive PCR results. These results again demonstrate the arbitrariness of the distinction between TB infection and disease in children.

It appears that PCR may have a useful but limited place in evaluating children for TB. A negative PCR result never eliminates TB as a diagnostic possibility, and a positive result does not confirm it. PCR's major use will be in evaluating children with significant pulmonary disease, when the diagnosis is not easily established by clinical or epidemiologic grounds. PCR may be particularly helpful in evaluating immunocompromised children with pulmonary disease, although published reports of PCR performance in such children are lacking. PCR may also aid in establishing the diagnosis of extrapulmonary TB, though only rare case reports have been published. However, performing PCR on gastric aspirates is not a useful test to distinguish between TB infection and disease and should not be used for children with normal chest radiographs.

### Serology and Antigen Detection

Despite dozens of studies published over the past several decades, serology has found little place in the routine diagnosis of TB in adults or children. Several recent studies have used the enzyme-linked immunosorbent assay (ELISA) to detect antibodies to various purified or complex antigens of *M. tuberculosis* in children. Rosen (40) used mycobacterial sonicates in an ELISA on samples from 31 children with clinical TB and found a sensitivity of 26% and a specificity of 40%. This ELISA was influenced by recent BCG vaccination in children under 5 years of age. Barrera et al. (41) used an ELISA that detects antibodies to purified protein derivative and found a sensitivity of 51% for culture-positive pulmonary TB cases in children, but the sensitivity was only 28% for the clinical cases. Hussey et al. (42) used an autoclaved suspension of *M. tuberculosis* to detect antibodies in serum from 132 children with clinical pulmonary TB; the test was 62% sensitive and 98% specific. Higher sensitivity was obtained among patients with positive culture results (69%, n = 35), miliary TB (100%, n = 6), tuberculous meningitis (80%, n = 15), and pleural effusion (78%, n = 16). No correlation was observed with the tuberculin skin-test result, BCG vaccination, or nutritional status whereas duration of therapy, increasing age and chronicity of infection were positively correlated. Delacourt et al. (43) used an ELISA to detect IgG and IgM antibodies directed against mycobacterial antigen A60 in children with TB. At a chosen specificity of 98%, IgG was detected in 68% of children with clinical disease when results were highly controlled for age and prior BCG vaccination. IgM detection had only a 19% sensitivity. However, using the same anti-A60 ELISA at a defined specificity of 95%, Turneer et al. (44) found the IgG sensitivity to be 26% for past TB, 6% for asymptomatic primary

TB, 14% for symptomatic TB, and 9% for NTM adenitis. No available serodiagnostic test for TB has adequate sensitivity, specificity, or reproducibility under various clinical conditions to be useful for diagnosing TB in children.

Mycobacterial antigen detection has been evaluated in clinical samples from adults, but rarely from children (45,46). Two recent assays detecting *M. tuberculosis*-specific antigens yielded high sensitivity and specificity in various clinical specimens from adults with TB (47,48). Measurement of tuberculostearic acid, a mycobacterial mycolic acid, has been used to detect *M. tuberculosis* in clinical specimens (49). Brooks et al. (50) demonstrated a sensitivity of 95% and specificity of 91% when chromatographic profile of carboxylic acids and detection of tuberculostearic acid were combined and compared with culture results and clinical findings in adults with pulmonary TB; however, these techniques require technically advanced equipment and expertise, which are not available where TB in children is most common. Their sensitivity and specificity in children are unknown.

### Implications for HIV Infection and Drug Resistance

The resurgence of TB over the last decade has coincided with the HIV pandemic. HIV-infected infants and children are in close contact with their caregivers, who may be infected with HIV and *M. tuberculosis* and are at high risk of developing infectious TB as they become immunocompromised. None of the available diagnostic tests for TB infection or disease in children has been evaluated systematically in children with HIV infection and pulmonary disease or suspected TB. In adults with HIV infection and TB, the sensitivity of diagnostic tests that rely on the host immune response, such as the TST or serology, is much lower than in nonimmunocompromised TB patients. It is likely that the tests' sensitivity also will be lower in HIV-infected children with TB. Tests that directly detect *M. tuberculosis*, such as PCR or antigen detection assays, may be particularly important for HIV-infected children. The culture yield of *M. tuberculosis* from children with HIV infection and TB is unknown but appears to be similar to that from non-HIV-infected children. The most important diagnostic clue for detecting TB in HIV-infected children is a history of contact with an adult who has infectious TB. Since TB may not have yet been diagnosed in this adult, a rapid and aggressive evaluation for TB in adults who care for the child is a critical part of the evaluation of the child.

The current prevalence of drug resistance among *M. tuberculosis* isolates in the United States is 8% to 14% (51, 52). Drug resistance is most common in patients who received treatment, are not responding to therapy, do not adhere to treatment, live in developing countries, are immunocompromised, are prisoners, are homeless, or are children exposed to adults at increased risk for drug resistance. Drug-resistant TB has increased significantly among children (52). Because of low culture yields from children with TB, the clinician must often rely on the antimicrobial susceptibility results for the *M. tuberculosis* isolate obtained from the adult source case who presumably infected the child. This again emphasizes the crucial need to identify and evaluate the source case for every child with TB. The rapid identification of drug-resistant organisms is necessary for control of drug-resistant TB. Various new methods, such as high-performance liquid chromatography or PCR and DNA sequence analysis, may help to identify and test for antimicrobial susceptibility within a few days of diagnosis, but these techniques remain experimental.

### Summary

Most recently developed sensitive and specific diagnostic tests have not found a place in the routine evaluation of children with suspected TB. Clinical criteria, particularly skin-test results, radiographic changes, and documented exposure to an infectious adult remain standard diagnostic methods. In industrialized countries, the local public health entity is a crucial partner to the clinician in establishing the diagnosis in the child and determining if drug resistance is present. As new diagnostic tests are developed, they must be evaluated against clinical criteria. The basic differences in pathophysiology of TB in adults and children must be considered before new tests are applied in pediatrics. It will be crucial to study the new techniques in children and not simply extrapolate from results for adults with TB.

*Dr. Khan is a postgraduate fellow in pediatric infectious diseases at Baylor College of Medicine, Houston, Texas. Dr. Starke is an associate professor at Baylor College of Medicine and current chairman of*

*CDC's Advisory Committee for the Elimination of Tuberculosis.*

### References

1. Starke JR, Correa AG. Management of mycobacterial infection and disease in children. Pediatr Infect Dis J 1995;14:455-70.
2. Styblo K, Rouillon A. Tuberculosis in developing countries: burden, intervention and cost. Bull Int Union Against Tuber Lung Dis 1990;65:6-24.
3. Starke JR, Jacobs R, Jereb J. Resurgence of tuberculosis in children. J Pediatr 1992;120:839-55.
4. Cantwell M, Snider D Jr, Cauthen G, Onorato I. Epidemiology of tuberculosis in the United States, 1985 through 1992. JAMA 1994;272:535-9.
5. Kochi A. The global tuberculosis situation and the new control strategy of the World Health Organization. Tuber Lung Dis 1991;72:1-6.
6. Raviglione MC, Snider DE, Kochi A. Global epidemiology of tuberculosis. Morbidity and mortality of a worldwide epidemic. JAMA 1995;273:220-6.
7. Dolin P, Raviglione M, Kochi A. Global tuberculosis incidence and mortality during 1990-2000. Bull World Health Organ 1994;72:213-20.
8. Barnes, PF Borrows, SA. Tuberculosis in the 1990s. Ann Intern Med 1993;119:400-10.
9. Hsu KHK. Contact investigation: a practical approach to tuberculosis eradication. Am J Public Health 1963;53:1761-9.
10. Nolan RJ Jr. Childhood tuberculosis in North Carolina: a study of the opportunities for intervention in the transmission of tuberculosis to children. Am J Public Health 1986;76:26-30.
11. Brailey ME. Tuberculosis in white and negro children. II. The epidemiologic aspects of the Harriet Lane study. Cambridge, MA: Harvard University Press, 1958.
12. Steiner P, Rao M, Victoria MS, et al. Persistently negative tuberculin reactions: their presence among children culture positive for *M. tuberculosis*. Am J Dis Child 1980;134:747-50.
13. Starke JR, Taylor-Watts KT. Tuberculosis in the pediatric population of Houston, Texas. Pediatrics 1989;84:28-35.
14. Vallejo J, Ong LT, Starke JR. Clinical features, diagnosis and treatment of tuberculosis in infants. Pediatrics 1994;94:1-7.
15. Lifschitz M. The value of the tuberculin skin test as a screening test for tuberculosis among BCG-vaccinated children. Pediatrics 1965;36:624-7.
16. Landi S, Ashley MJ, Grzybowski S. Tuberculin sensitivity following the intradermal and puncture methods of BCG vaccination. Can Med Assoc J 1967;97:222-5.
17. Joncas JH, Robitaille R, Gauthier T. Interpretation of the PPD skin test in BCG-vaccinated children. Can Med Assoc J 1975;113:127-8.
18. Johnson H, Lee B, Kelly E, McDonnell T. Tuberculin sensitivity and the BCG scar in tuberculosis contacts. Tuber Lung Dis 1995;35:113-7.
19. Menzies R, Vissandjee B. Effect of bacille Calmette-Guerin vaccination on tuberculin reactivity. Am Rev Respir Dis 1992;141:621-5.
20. Sepulveda RL, Burr C, Ferrer X, Sorensen RU. Booster effect of tuberculin testing in healthy 6-year-old school children vaccinated with bacille Calmette-Guerin at birth in Santiago, Chile. Pediatr Infect Dis J 1988;7:578-82.
21. American Thoracic Society. Diagnostic standards and classification of tuberculosis. Am Rev Respir Dis 1990;142:725-35.
22. American Academy of Pediatrics Committee on Infectious Diseases. Screening for tuberculosis in infants and children. Pediatrics 1994;93:131-4.
23. Schaaf HS, Beyers N, Gie RP, et al. Respiratory tuberculosis in childhood: the diagnostic value of clinical features and special investigations. Pediatr Infect Dis J 1995;14:189-94.
24. Parisi MT, Jensen MC, Wood BP. Pictorial review of the usual and unusual roentgen manifestations of childhood tuberculosis. Clin Imag 1994;18:149-54.
25. Delacourt C, Mani TM, Bonnerot V, et al. Computed tomography with normal chest radiograph in tuberculous infection. Arch Dis Child 1993;69:430-2.
26. Strumpf IJ, Tsang AY, Syre JW. Reevaluation of sputum staining for the diagnosis of pulmonary tuberculosis. Am Rev Respir Dis 1979;119:599-602.
27. Lipsky BA, Bates J, Tenover FC, Plorde JJ. Factors affecting the clinical value of microscopy for acid-fast bacilli. Rev Infect Dis 1984;6:214-22.
28. Kent PT, Kubica GP. Public health mycobacteriology – a guide for the level III laboratory. Atlanta, GA; Centers for Disease Control, 1985.
29. Abadco DL, Steiner P. Gastric lavage is better than bronchioalveolar lavage for isolation of *Mycobacterium tuberculosis* in childhood tuberculosis. Pediatr Infect Dis J 1992;11:735-8.
30. Carr DT, Karlson AG, Stillwell AA. A comparison of cultures of induced sputum and gastric washings in the diagnosis of tuberculosis. Mayo Clinic Proc 1967;42:23-5.
31. Chan S, Abadco DL, Steiner P. Role of flexible fiberoptic bronchoscopy in the diagnosis of childhood endobronchial tuberculosis. Pediatr Infect Dis J 1994;13:506-9.
32. Glidey Y, Hable D. Tuberculosis in childhood: an analysis of 412 cases. Ethiop Med J 1983;21:161-7.
33. Migliori AB, Borghesi A, Rossanigo P et al. Proposal for an improved score method for the diagnosis of pulmonary tuberculosis in childhood in developing countries. Tuber Lung Dis 1992;73:145-9.
34. Schluger NW, Rom WN. Current approaches to the diagnosis of active pulmonary tuberculosis. Am J Respir Crit Care Med 1994;149:264-7.
35. Eisenach KD, Sifford MD, Cane MD, Bates JH, Crawford JT. Detection of Mycobacterium tuberculosis in sputum samples using a polymerase chain reaction. Am Rev Respir Dis 1991;144:1160-3.
36. Noordhock A, Kolk A, Bjune G, et al. Sensitivity and specificity of polymerase chain reaction for detection of *Mycobacterium tuberculosis*: a blind comparison study among seven laboratories. J Clin Microbiol 1994;32:277-84.

37. Pierre C, Oliver C, Lecossier D, Bousssougant Y, Yemi P, Hance AJ. Diagnosis of primary tuberculosis in children by amplification and detection of mycobacterial DNA. Am Rev Respir Dis 1993;147:420-4.
38. Starke JR, Ong LT, Eisenach KD, et al. Detection of *M. tuberculosis* in gastric aspirate samples from children using polymerase chain reaction. Am Rev Resp Dis 1993;147(Suppl):A801.
39. Delacourt C, Poveda J-D, Churean C, et al. Use of polymerase chain reaction for improved diagnosis of tuberculosis in children. J Pediatr 1995;126:703-9.
40. Rosen EU. The diagnostic value of an enzyme-linked immunosorbent assay using adsorbed mycobacterial sonicates in children. Tubercle 1990;71:127-30.
41. Barrera L, Miceli I, Ritacco V, et al. Detection of circulating antibodies to purified protein derivative by enzyme-linked immunosorbent assay: its potential for the rapid diagnosis of tuberculosis. Pediatr Infect Dis J 1989;8:763-7.
42. Hussey G, Kibel M, Dempster W. The serodiagnosis of tuberculosis in children: an evaluation of an ELISA test using IgG antibodies to *M. tuberculosis*, strain H37RV. Ann Trop Paediatr 1991;11:113-8.
43. Delacourt C, Gobin J, Gaillard J-L, de Blic J, Veran M, Scheinmann P. Value of ELISA using antigen 60 for the diagnosis of tuberculosis in children. Chest 1993;104:393-8.
44. Turneer M, Nerom EV, Nyabenda J, Waelbroeck A, Duvivier A, Toppet M. Determination of humoral immunoglobulins M and G directed against mycobacterial antigen 60 failed to diagnose primary tuberculosis and mycobacterial adenitis in children. Am J Respir Crit Care Med 1994;150:1508-12.
45. Sada E, Ruiz-Palacios AM, Lopez-Vidal Y, et al. Detection of mycobacterial antigens in cerebrospinal fluid of patients with tuberculous meningitis by enzyme-linked immunosorbent assay. Lancet 1983;2:651-2.
46. Radhakrishnan VV, Sehgal S, Mathai A. Correlation between culture of *Mycobacterium tuberculosis* and detection of mycobacterial antigens in cerebrospinal fluid of patients with tuberculous meningitis. J Med Microbiol 1990;33:223-6.
47. Wadee AA, Boling L, Reddy SG. Antigen capture assay for detection of a 43-kilodalton *Mycobacterium tuberculosis* antigen. J Clin Microbiol 1990;28:2786-91.
48. Sada E, Aguilar D, Torres M, et al. Detection of lipoarabinomannan as a diagnostic test for tuberculosis. J Clin Microbiol 1992;30:2415-18.
49. Brooks JB, Daneshvar MI, Fast DM, et al. Selective procedures for detecting femtomole quantities of tuberculostearic acid in serum and cerebrospinal fluid by frequency-pulsed electron-capture gas-liquid chromatograph. J Clin Microbiol 1987;25:1201-6.
50. Brooks JB, Daneshvar MI, Harberger RL, et al. Rapid diagnosis of tuberculous meningitis by frequency-pulsed electron-captive gas-liquid chromatography detection of carboxylic acids in cerebrospinal fluid. J Clin Microbiol 1990;28:989-97.
51. Centers for Disease Control and Prevention. National action plan to combat multidrug-resistant tuberculosis. MMWR 1992;41:5-50.
52. Bloch AB, Cauthen GM, Onorato IM, et al. Nationwide survey of drug-resistant tuberculosis in the United States. JAMA 1994;271:665-71.
